# Postvention as Prevention: Coping with Loss at School

**DOI:** 10.3390/ijerph191811795

**Published:** 2022-09-19

**Authors:** Nikita Khalid, Nicole Zapparrata, Kevin Loughlin, Glenn Albright

**Affiliations:** 1 The Graduate Center, City University of New York, New York, NY 10016, USA; 2 Innovative Learning Sciences, Ascend Learning, Leawood, KS 66211, USA; 3 Baruch College, City University of New York, New York, NY 10010, USA

**Keywords:** postvention, trauma, mental health, simulation, professional development

## Abstract

Many Pre-K through grade 12 (PK-12) students have experienced traumatic events throughout the pandemic in a myriad of ways including the death of family members and peers, loss of social interaction and increased violence at home. The consequences can be traumatic and manifest themselves in fear, anxiety, anger, isolation, and loneliness. Too often this leads to depression, anxiety, grief, substance use disorders, post-traumatic stress disorder, suicidal ideation and even suicides. This study assesses the impact of an innovative virtual human role-play simulation that prepares PK-12 educators, administrators, and school staff to respond to a student death in the school community by creating communities of support to help manage traumatic loss. The simulation addresses crisis response planning, postvention plans, and provides learners with role-play practice in using evidence-based motivational interviewing communication strategies in conversations with students and colleagues after the occurrence of a death. The sample consisted of educators and staff who were recruited from geographically dispersed areas across the US between January 2021 through December 2021. Matched sample t-tests and ANOVAs were used to assess quantitative data, and a qualitative analysis software, MAXQDA, was used to assess open-ended response data. Results show statistically significant increases in school personnel’s preparedness and self-efficacy to recognize signs of trauma in their students and colleagues, and to approach them to talk about concerns and, if necessary, make a referral to support services. Simulations such as this hold tremendous potential in teaching educators how address trauma due to a student death.

## 1. Introduction

### 1.1. COVID-19 and Traumatic Loss

Throughout the COVID-19 pandemic, globally, individuals have been impacted by traumatic loss with increased exposure to grief, loss of social connectivity due to mandated quarantines and lockdowns and increases in suicidal ideation and suicides. COVID-19 has widened the gaps already existing in physical health disparities and contributed to declining mental health. Worldwide, approximately five million deaths occurred from the start of the pandemic, and 5.2 million children lost either a parent or a caregiver [1]. These estimates are based on global reports of COVID-related deaths, some of which come from countries unable to accurately report death rates; thus, the loss of life and corresponding impact on children and adolescents could be even greater. In the United States, COVID-19-related orphanhood resulting from caregiver death impacted over 140,000 children, with the rate of experiencing loss 4.5 times higher among children of racial and ethnic minority groups when compared with non-Hispanic white children [2]. Dependent on geographic region, COVID-19-related death of parents and caregivers is highest for Hispanic children, Black children, and for American Indian/Alaskan Native populations. Children who are orphaned by COVID-19 face adverse consequences such as poverty, abuse, and institutionalization [1].

According to the Centers for Disease Control and Prevention [3], experiencing loss during COVID-19 is similar to experiencing loss in other types of disasters or traumatic events. Grief occurs when there are disruptions to daily routines, loss of life, and any other way in which individuals feel that their stability has been compromised. Grief can include experiencing feelings of shock, anger, sadness or denial, heightened anxiety and distress, and changes in sleeping and eating patterns. Due to the heightened number of bereaved individuals associated with COVID-19, prolonged grief disorder is also a major concern [4]. Specifically, individuals experience more intense acute grief when deaths are associated with COVID-19 than when they are related to other natural illnesses not related to the pandemic [5,6]. The consequences of COVID-19 materialize in heightened rates of trauma, loss and grief for children and have long term impact [7]. In addition, because of higher bereavement rates, individuals are at a higher risk of psychological impairment, especially when experiencing separation distress, grief, and posttraumatic stress [8].

Multiple studies conducted globally demonstrate the negative outcomes of tragic loss, prolonged grief exposure, and heightened anxiety as a result of the pandemic. For example, a qualitative study conducted in Italy, one of the first COVID-19 hotspots with a dramatic rise in mortality, demonstrated that abandonment, anger, guilt, dehumanization, and rumination about the pandemic were key themes that emerged when participants were interviewed regarding their experiences losing family members during lockdown [9]. Participants indicated that online social support and connectivity were extremely valuable in helping them to process grief during this time.

### 1.2. The Mental Health Impact of Suicide and Bereavement during COVID-19

The pandemic has clearly affected youth mental health as evidenced by elevated levels of depression, stress, anxiety, and suicidal ideation. Prior to the pandemic, the rate of suicide for youth ages 10–24 was already high. This rate increased almost 60% from 2007 to 2018 and is the third leading cause of adolescent death for ages 15–19 [10]. In addition, youth suicide attempts and visits to the emergency department for self-harm have increased since the start of the pandemic [11]. Suicide risk increased when accounting for COVID-19-related post-traumatic stress [12], and for individuals with lower perceived social support [13]. Bereaved individuals who have experienced loss due to suicide and other pandemic-related deaths are at risk of developing complicated grief, or prolonged grief disorder, which is characterized by experiencing major difficulties in accepting the death of a significant other, family member, or close friend [14,15,16]. Studies have also linked COVID-19 bereavement to intensified psychological distress, especially for those individuals who had previous psychiatric diagnoses [17]. Altogether, COVID-19-related deaths, including those by suicide, have resulted in heightened instances of poor mental health, lack of social connectivity, increased presence of environmental stressors such as financial burden, and lack of access to support services. Thus, the pandemic has put us at the forefront of a mental health crisis where evidence-based approaches to address trauma, loss, grief, and suicide are important to implement in school communities.

### 1.3. Educator and Student Mental Health

Educators, being on the front lines, were impacted by the pandemic both personally and professionally. School closures have resulted in fewer student resources such as access to school counseling centers, afterschool activities, and tutoring services [18]. Educators experienced disruptions to their jobs with one of the biggest hurdles for many being learning how to teach and engage students through distance learning. This is a difficult transition due to student learning loss and the impact on social and emotional learning and the mental health needs of both students and educators. Due to quarantine, lockdowns, and restrictions on social gatherings during the pandemic, students and educators both experienced social isolation and lack of connection [19]. The only way in which people were able to interact was virtually, which was challenging the viability of technology.

Throughout the pandemic, many children reported increased levels of depression, anxiety, fatigue, and distress [20,21,22]. Some factors related to poor mental health outcomes in children included living in rural areas, having friends or family members in the healthcare field, knowing someone infected with the virus, and belonging to lower socioeconomic status households. In addition, trauma associated with the pandemic may have a lifelong impact on student learning, behavior, and student social, emotional, and psychological functioning [23]. Additionally, the impact of trauma required many children and adolescents to focus on basic needs such as safety and a sense of security rather than social and academic needs. This can lead to a higher likelihood of attention issues, lower cognitive functioning, behavioral problems, decreased school attendance and difficulty in social relationships [23,24,25,26].

### 1.4. Need for Postvention

Based on the amount of loss that society has experienced in the last several years due to the pandemic, it is important that researchers continue to examine the impact of crisis-response and other preventative programs on children and adolescents, especially those at risk for suicide. According to a survey conducted by the American Federation of Teachers and the New York Life Foundation [27], educators indicate that they would benefit from having more resources to support students’ social and emotional needs and that insufficient training is by far the top barrier to supporting grieving students. In total, 91% of educators stated that if there were bereavement training provided in their school or district, they would be interested in participating in it, while 92% stated that there should be a greater focus on supporting grieving students [27]. There is a clear desire from educators to be trained in grief and bereavement support for students, which underscores a critical need for postvention.

When responding to a death in the school community, whether it’s by suicide or related to COVID-19, we must address the social, emotional, and mental health consequences that includes creating a community of support amongst students, educators, and parents. This support is vital among bereaved individuals for loneliness is significantly associated with the probability of a post-bereavement suicide attempt and suicidal ideation [28]. This extends into social networks of support which are critical to promoting mental well-being and help students and educators in coping with traumatic loss. In addition, it is crucial to integrate evidence-based coping skills into prevention and postvention programs to promote protective factors and resilience. Evidence exists supporting postvention programs in augmenting mental health outcomes for those impacted by suicide, sudden death, and tragic loss [29]; however, there are gaps in the literature for postvention, as well as for the general use of trauma-informed approaches in schools not specific to suicide postvention [30]. Teaching educators postvention strategies through easily accessible online training where they can engage in active learning in applying motivational interviewing (MI) skills through role-play with virtual students holds great promise in augmenting postvention protocols and ultimately, supporting the student health and wellness.

### 1.5. Hypotheses

The objectives of this study were to examine the effectiveness of an online virtual role-play simulation designed to teach educators and staff to respond to a death in the school community. Specifically, we hypothesized that as a result of the simulation, as indicated by perceived preparedness and self-efficacy Likert-scales, participants would be better prepared and confident to (1) identify students in psychological distress, (2) talk to students about their concerns and allow them to open up about their feelings of loss, (3) make a referral to support services if necessary, and (4) motivate students and colleagues to engage in self-care. We also hypothesized, based upon Likert-scale measures, that the training would result in (1) high satisfaction ratings; (2) a decrease in stigma related to discussing suicide with others, and (3) an increase in self-reported gatekeeper behaviors two months following completion of the training that include identifying students in psychological distress, helping them to open up to talk about their feelings of loss, and connecting them to support services.

## 2. Materials and Methods

### 2.1. Design and Measures

The study followed a repeated measures design with pre-, post, and two-month follow-up surveys and was based on Kirkpatrick’s training evaluation model [31,32]. Kirkpatrick’s model comprises four levels: (1) satisfaction, (2) learning, impact on attitudes, knowledge, and/or skills (3) behavior changes, and (4) results including the long-term benefits derived from the program or intervention such as shifts in school mental health culture. Level two and level three are interconnected for improvements in skills, knowledge and especially changes in attitudes influence changes in behaviors. This study includes assessing the first three levels and not the fourth due to limitations in implementation and lack of accessibility of more global metrics such as school climate.

Level one satisfaction measures were assessed in the post survey and included:

1. Overall, how would you rate the training? (4-point Likert scale ranging from “poor” to “excellent”).
2. Would you recommend the training to your colleagues? (2-point Likert scale ranging from “Yes” to “No”).
3. Is the training based on scenarios relevant to you? (2-point Likert scale ranging from “Yes” to “No”).

Level one items also included school climate measures collected at post survey and were based on a 5-point Likert scale ranging from “Strongly Disagree” to “Strongly Agree”. They included items where participants were asked how much they disagree/agree with the following statements that began with:

If I apply the skills taught in the training:

- Student attendance will increase.
- Student academic success will improve.
- The school learning environment will become more supportive.
- Classroom safety will improve.
- My relationship with students will improve.

Level two measures were in the pre-, post, and follow-up surveys and included modified items from the validated Gatekeeper Behavior Scale (see Tables 2 and 3 for items) [33]. The Gatekeeper Behavior Scale (GBS) measures attitudes and intentions that have been shown to be related to changes in gatekeeper behaviors. This survey included two dimensions or subscales that were part of the original validity study: participant preparedness and self-efficacy to engage in gatekeeping behaviors. Lastly, three items measured perceived stigma assessing beliefs about suicide (see Table 4 for items).

Level three measures of behavior were measured at the two-month follow-up where participants were asked whether they believed that as a result of the training there were increases in the number of students: (1) identified as showing signs of psychological distress, (2) helped to open up about their feelings of loss, and (3) connected to support services. In addition, participants were also asked if as a result of the training, there were increases in the number of colleagues: (1) identified as showing signs of psychological distress, (2) helped to open up about their feelings of loss, and (3) connected to support services. Lastly, participants were asked if as a result of the training, there were increases in the number of conversations they have had with other teachers, staff and/or administrators (1) regarding students they were concerned about, and (2) about overall mental health in their school. Lastly, participants were asked to respond to the open-ended question “Now that you have completed the training, can you recall a situation where you used the skills learned in the training? Please describe what happened and be sure not to include any identifiable information.”

All participants agreed to an informed consent and then completed a pre-survey, then the 40 min simulation which was followed by the post and two-month follow-up surveys.

### 2.2. Methods

#### 2.2.1. Simulation Overview

**Figure 1:**
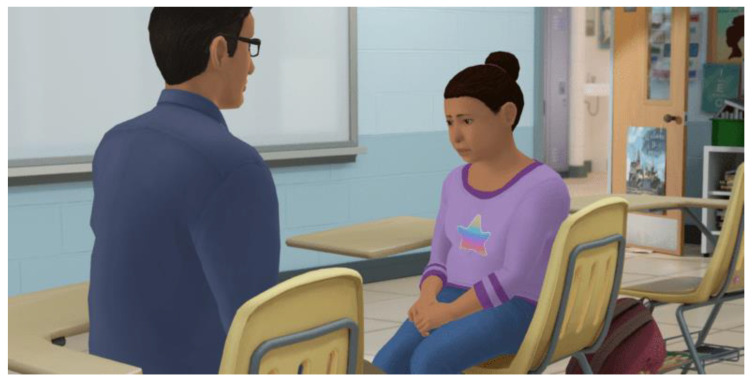
Screenshot from Resilient Together: Coping with Loss at School program.

*Resilient Together: Coping with Loss at School* is a virtual role-play simulation developed by Kognito (www.kognito.com). This simulation follows a similar learning methodology to other virtual role-play simulations that have demonstrated to be effective in training educators and staff in communication techniques that produce attitudinal and behavioral changes. For example, elementary school educators who were trained via simulated role-play reported an average increase of 25% in feelings of preparedness to recognize students in psychological distress and approach their parents to discuss referrals to support [34]. In this same study, educators reported a 36% increase in the number of students recognized as being in distress, a 54% in number of students with whom they had discussions about concerns, and a 72% increase in the number of parents with whom they had discussions about referrals to support for their children. Additional studies demonstrate similar findings for training via virtual role-play simulators [35,36,37]. In the *Resilient Together: Coping with Loss at School* simulation, participants enter an online environment where they practice role-playing with emotionally responsive intelligent virtual students coded with memory, personality, and will respond like real students who have experienced a loss. The 40 min simulation involves participants practicing role-playing one of two conversations, dependent on their students’ grade levels, one is with a virtual student and the other with a virtual teacher. These virtual humans model behaviors that school personnel often see during a highly sensitive time such as after a student suicide in the school community. A virtual coach provides ongoing feedback on effective and ineffective communication strategies and though practicing the role-plays, participants learn how to support students impacted by a death and support their colleagues experiencing compassion fatigue. Several studies have demonstrated the efficacious impact of role-play simulations that have implemented similar learning models as the one examined in this study [34,35,36,37]. A more detailed description of simulation design and learning methodology can be found in Albright et al. [38] as well as an overview on simulations in PK-12 [39]. Figure 1 shows a screenshot of the simulated role-play.

#### 2.2.2. Sampling and Sample Demographics

The sample initially consisted of 4500 educators and staff who were recruited from geographically dispersed areas across the US between January 2021 through December 2021 from district superintendent offices, principals, and by word-of-mouth. Participants gained free access to the simulation via institutional licenses purchased directly from the vendor by school districts or by state departments of education, health or public health, and mental health organizations, and could take the simulation at a time of their choosing and in a convenient location such as their home or office. Participants first completed the pre-survey followed by the simulation, then a post survey and two months later, a follow-up survey. Overall, the final sample size was 383 participants who completed all three surveys. Participants were able to opt out of any survey question they did not want to complete, including demographic information; therefore, demographic information is listed for only those participants who chose to fill out the information. Participants were primarily white female teachers. Table 1 provides complete demographic information. The average age of the sample was 51 years, with 62% being teachers, 12% staff members, 10% administrators, and the remainder of the sample consisting of mental health specialists (3.1%) and paraprofessionals (3.4%).

#### 2.2.3. Analyses

To determine whether preparedness and self-efficacy increased overtime as a result of the intervention, a series of one-way repeated measures ANOVAs were run on each item. Post hoc tests using a Bonferroni correction were used to make pairwise comparisons between pre-test and post-test means, pre-test and follow-up means, and post-test and follow-up means. Partial eta squared was calculated for each repeated measures ANOVA as a measure of effect size for each item to determine the magnitude of the effect of the training over time. Frequencies were calculated for satisfaction measures, level two measures regarding application of the skills learned in the training, and behavioral measures.

To incorporate qualitative data, MAXQDA 2020 software was used. This software combines quantitative processing with manual coding. We were able to thematically code the qualitative data and produce frequency tables to directly illustrate the research findings. The qualitative analysis involved coding for reoccurring themes using a joint inductive-deductive coding process. This process involved two independent coders, with each coder reading through responses individually and identifying common themes with the MAXQDA 2020 software. Once a final coding template was established by both coders independently, both coders coded the responses into the full set of thematic categories, refining themes based on overlapping categories. The coders resolved any discrepancies through discussion with one another and reported all thematic categories and the frequency of responses for those categories. Some statements could fit into multiple themes, thus percentages reported do not add to 100%. Statements have been copied directly as reported, without correcting for typos.

## 3. Results

### 3.1. Level One Satisfaction Measures

Overall, participants found the simulation to be very effective with 98% stating it was good (37.9%), very good (41.3%), or excellent (18.8%) and 92% indicating that they would recommend the simulation to colleagues. A total of 82% of participants indicated that the training is based on relevant scenarios.

### 3.2. Level Two Measures

There were significant mean Likert-scale increases (see Table 2, Table 3 and Table 4 for means and *p*-values)) from pre- to post-survey and pre- to follow-up survey in preparedness and self-efficacy to (1) recognize when a student is showing signs of psychological distress (such as being anxious, depressed or disengaged), (2) recognize changes in behavior in response to a loss (such as losing interest in activities, declines in grades or social isolation), (3) help a student open up to talk about their feelings of loss, (4) motivate a student to connect with support services, (4) motivate a colleague to connect with support services, and (5) engage in self-care (such as supporting a healthy mindset, acknowledging your emotions or maintaining close relationships), (6) motivate a colleague to engage in self-care, and (7) motivate a colleague to connect with support services. Partial eta squared (η_p_^2^; effect size measure) is reported in Table 2, Table 3 and Table 4 for each item to demonstrate the magnitude of the effect of the training over time. This effect size scale indicates 0.01 as a small effect, 0.06 as a medium effect, and 0.14 as a large effect.

There were also significant decreases from pre- to post-survey, and pre-to follow-up survey (*p* < 0.01) in the beliefs that (1) talking about suicide will increase the risk of suicide, and significant decreases from pre-test to post-test in the idea that (2) talking about suicide with someone who has lost a family member or friend by suicide should be avoided (*p* < 0.01). These measures had medium effect sizes. Table 2, Table 3 and Table 4 present the quantitative results.

Participants also either agreed or strongly agreed in the post-survey that if they apply the skills taught in the simulation, there would be increases in (1) student academic success = 72%), (2) student attendance = 64%, (3) classroom safety = 82%, (4) the school learning environment will become more supportive = 91%, and (5) relationships with students will improve = 90%.

### 3.3. Level Three Behavior Measures

Participants self-reported reported in the two-month follow-up survey that as a result of applying the skills they had learned in the training, they either agreed or strongly agreed that: (1) student attendance increased = 32%, (2) student academic success improved = 36%, (3) the school learning environment became more supportive = 61%, (4) classroom safety improved = 57%, and (5) their relationship with students improved = 61%. Additionally, respondents reported that as a result of taking the training, there has been an increase in the number of students and colleagues that they identified as showing signs of psychological stress (students = 24%; colleagues = 21%), helped open up to talk about their feelings of loss (students = 32%; colleagues = 28%), and connected to support services (students = 27%; colleagues = 17%).

### 3.4. Qualitative Measures

At the two-month follow-up, participants were also asked to respond to the question “Now that you have completed the training, can you recall a situation where you used the skills learned in the training? Please describe what happened and be sure not to include any identifiable information.”

A total of 348 open response comments were examined for this analysis. Respondents may have provided multiple themes if they discussed utilizing more than one skill during the incident they wrote about. Qualitative analysis was conducted via MAXQDA 2020, a software designed to streamline the process of coding qualitative data into useful themes. The coding process was primarily manual; however, the MAXQDA 2020 software assisted the qualitative coding process through key word lexical search capabilities and color coding after themes were identified by researchers. Frequency counts and proportions of responses that were related to each theme were created.

First, the manual iterative coding process was completed by two researchers separately, after which results were combined to reflect the themes which emerged. Results from both researchers were compared using reliability analysis in a two-way mixed model. Intraclass Correlation Coefficient (ICC) was used to determine the level of agreement between the two researchers. The ICC for this qualitative analysis was 0.996, indicating a high level of agreement between the researchers.

Results of the qualitative analysis revealed that a large portion of respondents had not had a chance to utilize the skills learned in the training or virtual instruction prevented them from doing so. However, of those who did, the most mentioned themes included recognition of distress in students, discussing concerns with distressed students, ability to implement open communication, knowing when to refer counseling, recognition of distress in colleagues, dealing with students who lost a family member, and discussing student mental health concerns with parents. The full results of this analysis are displayed in Table 5 below.

## 4. Discussion

The main objective of this study was to examine the effectiveness of an online virtual role-play simulation designed to teach educators and staff to respond to a death in the school community. We hypothesized that as a result of the simulation, participants would be better prepared and confident to respond to death in the school community.

Quantitative results demonstrated that *Resilient Together: Coping with Loss at School* was an effective tool for teaching learners how to recognize signs of psychological distress, and improved learners’ preparedness and self-confidence in their ability to engage in conversations with students and colleagues regarding support services. These findings align with findings from similar studies that assess the efficacy of simulated virtual role-play programs, specifically in significantly increasing attitudes regarding recognition, approach, and referral behaviors [34,35,36,37,38,39]. However, this study differs in that it also incorporates the importance of identifying the impact of loss and grief on school communities, differing from typical interventions that aim to improve mental health awareness. An important finding to note is that not only did the program assist learners in helping others, but it also increased their preparedness and confidence in their own self-care. The self-care component provides additional support for simulated role-play that previous papers do not. Coping with loss is difficult in any context, especially the death of loved ones. Our school communities have been heavily impacted by loss due to COVID-19 and are experiencing prolonged effects of stress and loss in students’ and educators’ reintegration into the academic environment. In addition, because the program resulted in significant mental health and suicide stigma reduction, this could help lead to potential changes in institutional climate toward greater acceptance of having conversations about mental health and suicide, thus creating a safer and more trusting educational environment.

When asked about using the skills learned in the simulation, participants reported instances of recognizing distress in students, colleagues, and other school community members. Additionally, they reported increases in approaching and referring students in distress to support services. Lastly, participants mentioned how helpful the program was specifically in helping others deal with the loss of family members or other members of the school community. For specific frequency of the occurrence of these behaviors, please see the qualitative data reported in Table 5. The qualitative data show that participants were able to better understand the impact of simulated role-plays in training school community members how to recognize those in distress and how to engage them in conversations to augment their behavioral health as well as create communities of support within their schools. The benefits of building rapport between students and teachers, and between teachers and administrators themselves cannot be overstated due to potential impact of fostering a positive and safe school climate.

Lastly, many respondents, nearly half in the qualitative data, mentioned that they have not had a chance to utilize the skills learned in the training due to needing to take necessary precautions because of the pandemic as well as virtual classroom instruction. At the same time, a substantial number of respondents reported that they utilized the skills practiced. This observation is important as many school settings are still taking measures to protect students from COVID-19 and ensuing co-variants. This study provides evidence that the use of virtual role-play simulations such as *Resilient Together: Coping with Loss at School* are an effective approach to augmenting prevention and postvention programs within school districts that are vital in supporting student and faculty mental health and well-being.

## 5. Limitations

As in many field studies, recruitment of subjects for experimental and control groups through random assignment was not possible. Thus, one limitation is that we implemented an experimental design that examined within-group differences that did not include a control group. Another limitation is that behavioral data were self-reported. Ideally, we would have preferred access to school records on classroom attendance, student academic performance, and incidences of school safety, etc. Additionally, the data collected in this study are specific to educator and staff perceptions of student mental well-being; thus, incorporating student data would have allowed us to further assess the efficacy of the intervention. Another limitation was that although the items used to assess attitudinal change were based on the validated Gatekeeper Behavior Scale, the scale used in this study was not previously validated. Thus, although convergent validity has been established for the Gatekeeper Behavior Scale [33], since the scale used in this study was modified, future studies should examine the convergent validity of the modified scale. Lastly, to increase interrater reliability in the qualitative analysis, ideally, we would have liked more researchers to code responses to ensure accuracy. Given these limitations, the results reported in this paper are assumed to represent the population of those who participated in the *Resilient Together: Coping with Loss at School* program.

## 6. Conclusions

As evidenced in the postvention literature, there is an overwhelming need for school-based interventions. Adolescent and young adult populations are at the highest risk for suicide clusters and contagion effects. Without proper postvention protocol, it is difficult to address a death in a school community, particularly a death by suicide, which can increase the occurrence of suicide clusters and directly impact already at-risk students by amplifying negative emotions (e.g., feelings of hopelessness, anxiety, loneliness). The data from this study provide encouraging evidence that simulations such as *Resilient Together: Coping with Loss at School* can augment postvention efforts. The current use of online virtual role-plays to manage postvention conversations had a positive impact on the perceived preparedness and self-efficacy of educators and staff on their postvention efforts. Overall, the impact of death and the loss of loved ones effects the entire school community, and postvention techniques are essential in coping with the loss. The results from this study provide support that postvention efforts are strengthened through the use of virtual interventions that promote recognition, approach and referral behaviors and can be effective when helping school communities to cope with loss, which has been especially prominent during the COVID-19 pandemic.

## Figures and Tables

**Table 1:** Demographics.

Demographic Item	*n*	Percentage (%)
Female	331	86.4
Male	34	8.9
Non-binary	1	0.3
I prefer not to answer	15	3.9
I prefer to self-describe	0	0
American Indian/Alaska Native	13	3.4
Asian	12	3.1
Black/African American	28	7.3
Hispanic/Latinx	64	16.7
Native Hawaiian/other Pacific Islander	1	<0.1
White	240	62.7
I prefer not to answer	96	25.1
Teaching	238	62.1
Administrator	38	9.9
Mental Health Specialist	12	3.1
Staff Member	47	12.3
Paraprofessional	13	3.4
School resource officer	0	0
Other	33	8.6
Pre-K	97	25.3
Elementary School	156	40.7
Middle/Junior High/Intermediate School	62	16.2
High School	29	7.6
Other	2	0.5

**Table 2:** In response to a real or possible death in your school or community, please indicate your preparedness to.

Measure	Pre-Test Mean (SD)	Post-Test Mean (SD)	Follow-Up Mean (SD)	*n*	Repeated Measures ANOVA (η_p_^2^ in Parenthesis)	Significant Time Points
Recognize when a student is showing signs of psychological distress	3.57 (0.767)	4.03 (0.648)	3.81 (0.686)	381	72.8 *** (0.161)	All
Recognize changes in behavior in response to the loss	3.68 (0.749)	4.08 (0.623)	3.90 (0.663)	381	61.8 *** (0.140)	All
Help a student open up to talk about their feelings of loss	3.53 (0.751)	4.02 (0.669)	3.73 (0.693)	376	80.4 *** (0.177)	All
Motivate a student to connect with support services	3.55 (0.797)	4.06 (0.661)	3.79 (0.716)	378	87.4 *** (0.188)	All
Engage in self-care	3.57 (0.771)	4.05 (0.657)	3.83 (0.709)	379	79.2 *** (0.173)	All
Motivate a colleague to engage in self-care	3.63 (0.778)	4.04 (0.687)	3.83 (0.717)	379	56.4 *** (0.130)	All
Motivate a colleague to connect with support services	3.59 (0.811)	4.02 (0.708)	3.77 (0.730)	376	55.9 *** (0.130)	All

Note. This note is relevant for the table above. Items were on a 5-point scale. *** *p* < 0.001, ** *p* < 0.01, * *p* < 0.05, ns = not significant. Items were scored using a scale from “Very Low = 1” to “Very High = 5”.

**Table 3:** In response to a real or possible death in your school or community, please indicate how much you disagree/agree with the following statements that begin with “I feel confident in my ability to”.

Measure	Pre-Test Mean (SD)	Post-Test Mean (SD)	Follow-Up Mean (SD)	*n*	Repeated Measures ANOVA (η_p_^2^ in Parenthesis)	Significant Time Points
Recognize when a student is showing signs of psychological distress	3.93 (0.630)	4.20 (0.555)	4.08 (0.613)	362	28.4 *** (0.073)	All
Recognize changes in behavior in response to the loss	3.97 (0.604)	4.21 (0.545)	4.11 (0.576)	359	25.5 *** (0.067)	All
Help a student open up to talk about their feelings of loss	3.85 (0.667)	4.18 (0.562)	3.99 (0.623)	357	39.8 *** (0.101)	All
Motivate a student to connect with support services	3.84 (0.701)	4.18 (0.585)	3.98 (0.636)	361	44.9 *** (0.111)	All
Engage in self-care	3.86 (0.663)	4.17 (0.599)	3.99 (0.666)	362	34.2 *** (0.086)	All
Motivate a colleague to engage in self-care	3.88 (0.674)	4.18 (0.608)	4.02 (0.637)	361	36.3 *** (0.177)	All
Motivate a colleague to connect with support services	3.86 (0.676)	4.17 (0.613)	3.99 (0.673)	356	33.0 *** (0.085)	All

Note. This note is relevant for the table above. Items were on a 5-point scale. *** *p* < 0.001, ** *p* < 0.01, * *p* < 0.05, ns = not significant. Items were scored using a scale from “Strongly Disagree = 1” to “Strongly Agree = 5”.

**Table 4:** Please indicate if you disagree/agree with the following statements.

Measure	Pre-Test Mean (SD)	Post-Test Mean (SD)	Follow-Up Mean (SD)	*n*	Repeated Measures ANOVA (η_p_^2^ in Parenthesis)	Significant Time Points
Talking to a student about suicide will increase the risk that the student will contemplate suicide	2.28 (0.844)	1.97 (0.968)	2.06 (0.831)	354	18.5 *** (0.050)	Pre-Post Pre-FU
When a person dies by suicide, most people feel that it is better not to talk about it with that person’s family or friends	2.67 (1.01)	2.73 (1.04)	2.75 (1.01)	347	NS (0.003)	None
I would personally avoid talking about suicide with someone who has lost a family member or friend by suicide	2.55 (0.950)	2.36 (0.886)	2.36 (0.863)	347	25.5 *** (0.069)	All

Note. This note is relevant for the table above. Items were on a 5-point scale. *** *p* < 0.001, ** *p* < 0.01, * *p* < 0.05, ns = not significant. Items were scored using a scale from “Strongly Disagree = 1” to “Strongly Agree = 5”.

**Table 5:** Now that you have completed the training, can you recall a situation where you used the skills learned in the training? Please describe what happened and be sure not to include any identifiable information.

Theme	Frequency (Researcher 1)	Frequency (Researcher 2)	Delta
No situation occurred	156	156	0
Recognition of distress in students	45	39	6
Discussing concerns with distressed students	38	35	3
Ability to implement open communication	34	34	0
Referral to counseling	33	27	6
Recognition of distress in colleagues	27	33	6
Unable to implement skills due to COVID/virtual instruction	23	25	2
Student loss of a family member	21	21	0
Discussions with parents/caregivers for students in distress	17	14	3
Implementation of mental health discussions with colleagues	13	11	2
Implementation of mental health support communities	11	5	6
Ability to help to cope with COVID-related hardships	11	12	1
Loss of a student	8	8	0
Did not find training useful	7	7	0
Feel better prepared to help	7	7	0
Not applicable due to role	5	3	2
Ability to help to cope with ailing/ill family members	4	2	2
Already learned prior to the training due to role	4	0	4
Loss of a staff member/colleague	3	3	0
Does not want to disclose information	3	3	0
Recognition of distress in a parent/caregiver	3	0	3
Recognition of family members in psychological distress	2	5	3
Student suicide	1	0	1
Used the skills learned for self-help	1	1	0
Intervention and stopping violent student behavior	1	1	0

## Data Availability

Restrictions apply to the availability of these data. Data were obtained from Ascend Learning and are available from glenn.albright@baruch.cuny.edu with the permission of Ascend Learning.
